# Noninvasive In Vivo Thrombus Imaging in Patients With Ischemic Stroke or Transient Ischemic Attack—Brief Report

**DOI:** 10.1161/ATVBAHA.122.318204

**Published:** 2023-07-13

**Authors:** Beth Whittington, Evangelos Tzolos, Rong Bing, Jack Andrews, Christophe Lucatelli, Mark G. MacAskill, Adriana A.S. Tavares, Tim Clark, Nicholas L. Mills, Jennifer Nash, Damini Dey, Piotr J. Slomka, Norman Koglin, Andrew W. Stephens, Edwin J.R. van Beek, Colin Smith, Marc R. Dweck, Michelle C. Williams, William Whiteley, Joanna M. Wardlaw, David E. Newby

**Affiliations:** BHF Centre for Cardiovascular Science (B.W., E.T., R.B., J.A., M.G.M., A.A.S.T., N.L.M., J.N., E.J.R.v.B., M.R.D., M.C.W., D.E.N.), University of Edinburgh, United Kingdom.; Centre for Clinical Brain Sciences (W.W., J.M.W.), University of Edinburgh, United Kingdom.; Usher Institute (N.L.M.), University of Edinburgh, United Kingdom.; Division of Pathology (C.S.), University of Edinburgh, United Kingdom.; UK Dementia Research Institute Centre (J.M.W.), University of Edinburgh, United Kingdom.; Edinburgh Imaging, Queen’s Medical Research Institute, United Kingdom (C.L., M.G.M., A.A.S.T., T.C., E.J.R.v.B., M.R.D., M.C.W., D.E.N.).; Life Molecular Imaging GmbH, Berlin, Germany (N.K., A.W.S.).; Department of Medicine, Division of Artificial Intelligence in Medicine, Biomedical Imaging Research Institute, Cedars-Sinai Medical Centre, Los Angeles, CA (D.D., P.J.S.).

**Keywords:** autoradiography, ischemic stroke, positron emission tomography, thromboembolism, thrombosis

## Abstract

**BACKGROUND:**

^18^F-GP1 is a novel positron-emitting radiotracer that is highly specific for activated platelets and thrombus. In a proof-of-concept study, we aimed to determine its potential clinical application in establishing the role and origin of thrombus in ischemic stroke.

**METHODS:**

Eleven patients with recent ischemic stroke (n=9) or transient ischemic attack (n=2) underwent ^18^F-GP1 positron emission tomography and computed tomography angiography at a median of 11 (range, 2–21) days from symptom onset. ^18^F-GP1 uptake (maximum target-to-background ratio) was assessed in the carotid arteries and brain.

**RESULTS:**

^18^F-GP1 uptake was identified in 10 of 11 patients: 4 in the carotid arteries only, 3 in the brain only, and 3 in both the brain and carotid arteries. In those with carotid uptake, 4 participants had >50% stenosis and 3 had nonstenotic disease. One case had bilateral stenotic disease (>70%), but only the culprit carotid artery demonstrated ^18^F-GP1 uptake. The average uptake was higher in the culprit (median maximum target-to-background ratio, 1.55 [interquartile range, 1.26–1.82]) compared with the contralateral nonculprit carotid artery (maximum target-to-background ratio, 1.22 [1.19–1.6]). In those with brain ^18^F-GP1 uptake (maximum target-to-background ratio, 6.45 [4.89–7.65]), areas of acute infarction on computed tomography correlated with brain ^18^F-GP1 uptake in 6 cases. Ex vivo autoradiography of postmortem infarcted brain tissue showed focal uptake corresponding to intraluminal thrombus within the culprit vessel and downstream microvasculature. There was also evidence of diffuse uptake within some of the infarcted brain tissue reflecting parenchymal petechial hemorrhage.

**CONCLUSIONS:**

^18^F-GP1 positron emission tomography and computed tomography angiography is a novel noninvasive method of identifying in vivo cerebrovascular thrombosis, which holds major promise in understanding the role and origin of thrombosis in stroke.

**REGISTRATION:**

URL: https://www.clinicaltrials.gov; Unique identifier: NCT03943966.

**Highlights**

- ^18^F-GP1 positron emission tomography and computed tomography identify thrombus on carotid plaques in patients with acute ischemic stroke or transient ischemic attack.
- Brain uptake of ^18^F-GP1 is seen in all areas of acute computed tomography and magnetic resonance imaging–defined infarction, corresponding to microvasculature intraluminal thrombus or diffuse petechial parenchymal hemorrhage.
- In 3 of the 11 cases ^18^F-GP1 positron emission tomography and computed tomography suggested an additional mechanism of the cause of stroke or transient ischemic attack.

Ischemic strokes are predominantly caused by thromboembolism or thrombosis of the cerebral arteries. However, the role and origin of thrombus in some stroke subtypes is poorly understood and debated, especially cryptogenic or lacunar strokes.^1^ Moreover, the origin of the thromboembolus cannot be reliably identified using current approaches because there are either multiple potential sources or no apparent source can be found.

Direct and specific noninvasive identification of in vivo thrombus has recently become possible using ^18^F-GP1 combined positron emission tomography (PET) and computed tomography (CT) angiography. ^18^F-GP1 is a positron-emitting radiotracer that binds with high selectivity and specificity to glycoprotein IIb/IIIa receptors on activated platelets. Preliminary clinical studies have demonstrated detection of in vivo arterial and venous thrombi in patients with pulmonary thromboembolic disease, acute coronary syndrome, and bioprosthetic aortic valves.^2–6^ To our knowledge, there has only been 1 published case of detection of arterial thrombus in acute cerebral infarction with ^18^F-GP1 uptake.^7^ In this proof-of-concept pilot study, we aimed to determine whether ^18^F-GP1 PET and CT angiography has the potential to establish the presence, location, and origin of thrombus in patients with recent ischemic stroke.

## METHODS

The data that support the findings of this study are available from the corresponding author upon reasonable request.

### Study Design and Study Population

This was a single-center prospective proof-of-concept study. Patients with transient ischemic attack or stroke were recruited from the Stroke Service in Edinburgh between January 2020 and February 2021. Inclusion criteria were age over 40 years with a recent (≤30 days) stroke or transient ischemic attack according to the American Heart and Stroke Association guidelines.^8^ Exclusion criteria included intracerebral hemorrhage, inability or unwillingness to give informed consent, inability to tolerate the supine position, estimated glomerular filtration rate <30 mL/min per 1.73 m^2^, allergy to iodinated contrast agents, severe significant comorbidity, and women who are pregnant or breastfeeding. A clinical stroke diagnosis was made and classified with the Oxford Community Stroke Project classification system.^9^ Patients underwent investigations including 12-lead electrocardiogram, 5-day electrocardiogram monitoring, carotid Doppler ultrasound, and brain imaging with either noncontrast CT or magnetic resonance imaging. The potentially symptomatic carotid side was defined by the clinical team based on symptoms/signs without knowledge of the PET–CT findings. Classification of stroke cause using the Trial of Org 10172 in Acute Stroke Treatment criteria was performed by clinicians blinded to the ^18^F-GP1 PET results.^10^ The study was approved by the South-East Scotland Regional Ethics Committee (18/SS/0143). All participants provided written informed consent.

### ^18^F-GP1 PET and CT

Combined ^18^F-GP1 PET and CT angiography was performed on a hybrid scanner (Biograph mCT; Siemens Medical Systems, Erlangen, Germany). PET list mode acquisition was performed 60 minutes after intravenous injection of a target dose of 250 MBq ^18^F-GP1 with 20-minute bed positions centered over the aortic arch, carotid arteries, and brain.^5^ Following CT attenuation correction, contrast-enhanced CT angiography of the cerebral and carotid circulation was performed (50 mL of 400 mg/mL at 5 mL/s, iomeron; Bracco, Milan, Italy). Helical CT was performed with tube voltage with tube current selected automatically based on scout images.

### Image Analysis

Images were analyzed using dedicated software (FusionQuant, version 1.21.0421; Cedars Sinai Medical Centre, Los Angeles, CA) as described previously.^4,5^ Before analysis, reconstructed PET images were fused with CT angiograms using rigid translation of PET images and alignment with points of reference, such as the bone marrow of the cervical vertebra and the blood pool of jugular veins and great vessels. Qualitative ^18^F-GP1 uptake was independently assessed by consultant radiologists (M.C.W., E.J.R.v.B., and J.M.W.). We took a systematic approach to quantitative assessments of PET uptake within the brain and carotid arteries. Within the brain, quantitative ^18^F-GP1 uptake was determined using 3-dimensional spherical volumes of interest centered on the region of interest adjusted to encompass the area of visual uptake to give maximum standardized uptake values. To determine the brain background activity, three 3-dimensional spherical volumes were placed over the contralateral noninfarcted brain tissue, and an average of maximum standardized uptake values was taken as described previously.^11,12^ Similarly, for the carotid uptake, the internal jugular vein at the level of the carotid bifurcation was used as the background blood pool activity and an average of three maximum standardized uptake values was taken as described previously.^13^ Uptake values were assessed using maximum standardized uptake values, mean standardized uptake values, maximum target-to-background ratio (TBR_max_), and mean target-to-background ratio. In the absence of carotid ^18^F-GP1 uptake, standardized uptake value measurements were taken at the largest atheromatous plaque within the carotid bifurcation or in the proximal 1 cm of the internal carotid artery if no plaque was present as described previously.^13^

### Statistical Analysis

Categorical variables were expressed as number (percentage) and continuous variables as mean±SD or median (interquartile range) if data were not normally distributed. Given the small sample size, no formal statistical testing was performed. The radiation exposure was calculated using the dose-length product and corresponding conversion factors (0.017 mSv/mGy.cm; attenuation correction, 0.013 mSv/mGy.cm; CT head, 0.024 mSv/mGy.cm; and test bolus and monitoring, 0.027 mSv/Gy.cm) and using 0.0186±0.0030 mSv/MBq ^18^F-GP1 to calculate the effective dose of the radiopharmaceutical (45-minute voiding and with ICRP [International Commission on Radiological Protection] 103).^14^

### Ex Vivo ^18^F-GP1 Binding to Human Thrombus

Postmortem brain tissue samples from patients with acute ischemic stroke and control patients (not experienced an ischemic stroke) were obtained from the Edinburgh Brain Bank with full ethical approval (21/ES/0087) for the proposed studies. ^18^F-GP1 autoradiography was performed on brain samples (n=12). See the Supplemental Material for further details.

## RESULTS

### Study Population

Participants (n=11 patients; 76±13 years; n=4 women) had high prevalence of hypertension and hypercholesterolemia (Table 1). Five participants had carotid endarterectomy, 2 had a potential cardioembolic cause (1 with permanent atrial fibrillation and inadequate anticoagulation and 1 with multiple previous cortical infarcts in different territories), and 4 had a stroke of undetermined etiology (Table 1; Table S1).

**Table 1.**
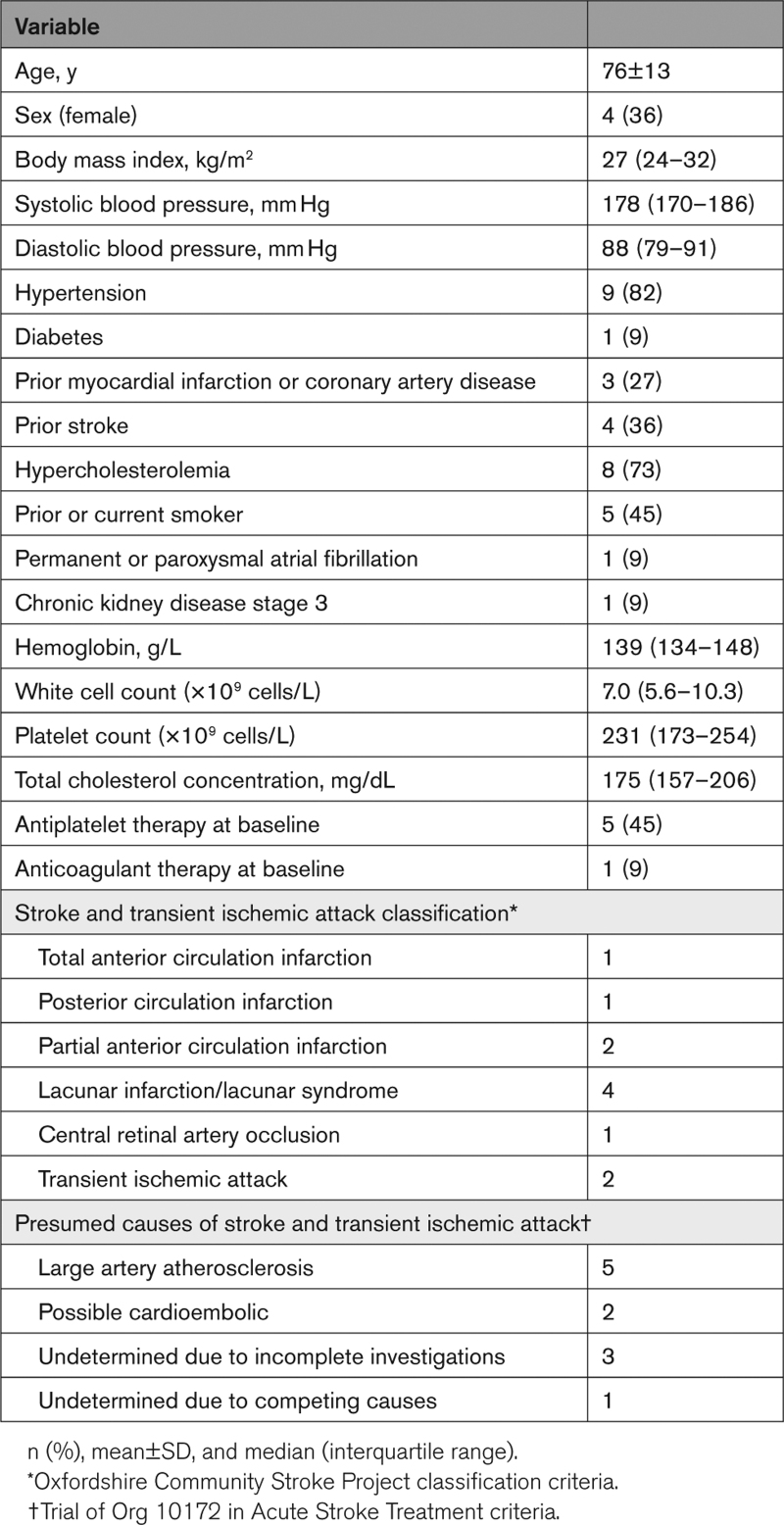
Baseline Characteristics of the Study Population and Classification and Cause of Stroke and Transient Ischemic Attack

### PET and CT Imaging

All participants underwent ^18^F-GP1 PET and CT at a median of 11 (range, 2–21) days from symptom onset. After an injection of 247±2 MBq of ^18^F-GP1, the standardized uptake value for the blood pool was 1.60±0.52 g/mL and for the brain parenchyma was 0.27±0.13 g/mL at 60 minutes. Average radiation exposure of patients from the ^18^F-GP1 injection was estimated at 4.6 mSv. The total overall radiation dose for ^18^F-GP1 PET and CT angiography per patient was 11.1±1.3 mSv.

Uptake of ^18^F-GP1 was identified in 9 of 11 cases: 3 in the carotid arteries only, 3 in the brain only, and 3 in both the brain and carotid arteries. In the 2 participants without carotid or brain ^18^F-GP1 uptake, 1 had a right central retinal artery occlusion and 1 had a right-sided retinal transient ischemic attack (Table S1).

### Carotid ^18^F-GP1 Uptake

Carotid ^18^F-GP1 uptake was observed in 7 patients either at the common carotid artery bifurcation or the proximal internal carotid artery in association with atheroma: TBR_max_, 1.70 (1.63–1.88). Two participants had <30% stenosis of ipsilateral carotid artery, 1 had 30% to 49% stenosis, 1 had 50% to 69% stenosis, and the remaining 3 had >70% stenosis. Of the 3 participants with >70% stenosis, 1 had >70% stenosis of the nonculprit internal carotid artery but without ^18^F-GP1 uptake: ipsilateral carotid artery TBR_max_, 3.15 compared with contralateral artery TBR_max_, 1.20 (Figure).

**Figure.**
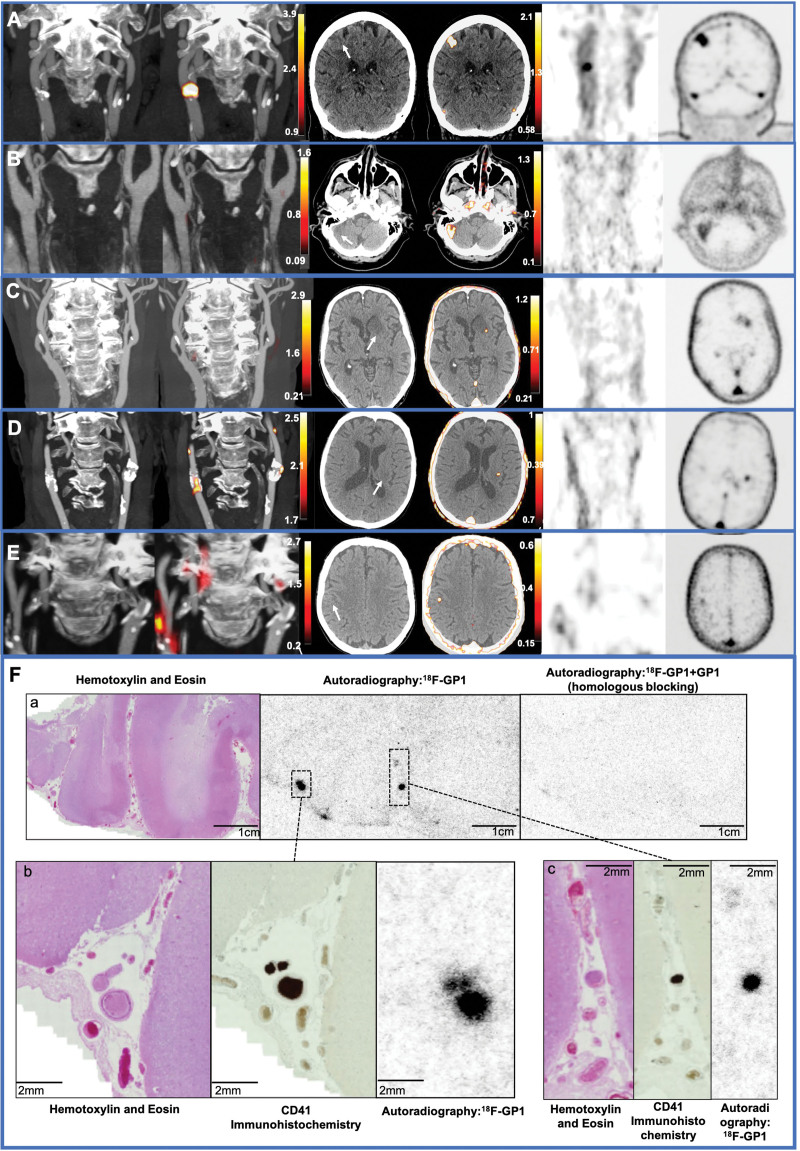
**Carotid artery and brain ^18^F-GP1 uptake. A** through **E**, First column: computed tomography angiogram of the carotid arteries. Second column: combined ^18^F-GP1 positron emission tomography (PET) and computed tomography angiogram of the carotid arteries. Third column: PET only carotid artery images. Fourth column: computed tomography angiogram of the brain. Fifth column: combined ^18^F-GP1 PET and computed tomography angiogram of the brain. Sixth column: PET only brain images. **A**, Patient presenting with left arm weakness with bilateral severe carotid stenosis with focal ^18^F-GP1 uptake (yellow/red; column 2) in the culprit right common carotid bifurcation (Table S1; patient number 2). Severe calcified and noncalcified plaque with severe stenosis of contralateral nonculprit internal carotid artery with no ^18^F-GP1 uptake. Right frontal lobe cortical infarction seen on computed tomography with diffuse ^18^F-GP1 uptake consistent with cortical infarction secondary to carotid thromboembolism. **B**, Right posterior circulation infarction with no uptake seen in the carotid arteries (column 2) with sagittal contrast-enhanced computed tomography angiography of the head showing area of acute infarction in the right cerebellum (arrow) and ^18^F-GP1 uptake encompassing the area of infarction (yellow/red; column 5; Table S1; patient number 6). There is uptake seen bilaterally in the cavernous internal carotid arteries, which represents background activity within the blood pool. **C**, Left striatocapsular (ie, large subcortical) infarct thought to be of cardioembolic origin with no uptake in carotid arteries with axial contrast-enhanced computed tomography angiography of the head showing an area of acute infarction in left basal ganglia (arrow) with focal ^18^F-GP1 uptake adjacent to the area of infarction (yellow/red; column 5; Table S1; patient number 8). **D**, Patient presenting with right upper limb weakness with bilateral atheroma at the common carotid artery bifurcations and bilateral ^18^F-GP1 uptake (yellow/red; column 2; Table S1; patient number 10). Sagittal contrast-enhanced computed tomography showing area of acute small subcortical infarction in the left corona radiata (arrow) with corresponding ^18^F-GP1 focal uptake. The cause of stroke was undetermined due to competing causes: small vessel disease and the presence of atheroma at carotid bifurcation on ipsilateral side to stroke symptoms without significant (30%–49%) stenosis. Carotid ^18^F-GP1 uptake suggests carotid atheroembolism. **E**, Patient presenting with left arm weakness, bilateral calcified carotid atheroma, and unilateral ^18^F-GP1 uptake at culprit right internal carotid artery (Table S1; patient number 1). There is a small cortical infarct in the posterior right frontal cortex (arrow) in area corresponding to stroke symptoms with ^18^F-GP1 uptake (yellow/red) consistent with cortical infarction secondary to carotid thromboembolism. In **A** through **E**, PET signal is seen within the dural sinuses, which represents background activity within the blood pool. **F**, ^18^F-GP1 uptake in ex vivo infarcted brain tissue. Postmortem infarcted brain tissue following acute middle cerebral artery territory ischemic stroke. **Fa**, Hematoxylin and eosin (H&E) staining showing infarcted tissue with autoradiography showing diffuse ^18^F-GP1 uptake representing microthrombi in vessels distal to the presumed primary site of arterial occlusion, which is blocked by coincubation with excess unlabeled GP1 reference standard to block the glycoprotein IIb/IIIa receptor. **Fb**, H&E staining showing a small vessel within the subarachnoid space, which shows prominent CD41 (platelet glycoprotein IIb) immunoreactivity consistent with thrombus, which and has corresponding intense focal ^18^F-GP1 uptake. **Fc**, H&E-stained section showing microthrombus within subarachnoid small vessels with corresponding CD41 staining and intense focal ^18^F-GP1 uptake.

Five participants had a stroke or transient ischemic attack due to carotid artery atherosclerotic stenosis and had carotid endarterectomy. Three of these had unilateral uptake, which corresponded to the clinically adjudicated carotid plaque. Two participants did not show uptake on culprit carotid plaque. Of these, 1 showed no carotid uptake in the context of a right retinal central artery occlusion although localized ^18^F-GP1 uptake was identified around their prosthetic aortic valve. The final participant showed unilateral carotid ^18^F-GP1 uptake in the contralateral vessel, which contained a previously implanted internal carotid artery stent.

Two participants had bilateral carotid ^18^F-GP1 uptake. The first had a small subcortical ischemic stroke attributed to small vessel disease although some carotid atheroma (40% stenosis) was present in the ipsilateral internal carotid artery. The second had a total anterior circulatory infarction treated with thrombolysis and clinically had been diagnosed with an undetermined origin of thromboembolism.

### Brain ^18^F-GP1 Uptake

Six participants had brain ^18^F-GP1 uptake: TBR_max_, 6.45 (4.89–7.65). All 6 participants had acute infarction demonstrable on brain imaging with ^18^F-GP1 uptake colocalizing to the area of infarction (Figure). Two cases showed focal ^18^F-GP1 uptake, which was visually smaller than, and adjacent to, the corresponding area of infarction: one within the left basal ganglia within the left corona radiata and the other in the posterior frontal cortex. Three cases showed qualitatively larger and more diffuse cortical regions of uptake, encompassing most or all of the area of infarction. The remaining case also showed visual uptake encompassing the entire area of cortical infarction, which was smaller in size but corresponded to an acute small cortical infarction.

One patient presented with symptoms of a posterior circulation stroke having had 2 previous ischemic strokes: a right middle cerebral artery infarction and a right thalamic stroke. The patient had previously undergone percutaneous patent foramen ovale closure for cryptogenic stroke 13 years ago. While there was clear cerebral uptake of ^18^F-GP1 within the right cerebellar infarct (Figure), there was no vascular, cardiac, or device-related ^18^F-GP1 uptake.

### ^18^F-GP1 Binding to Human Thrombus

Ex vivo autoradiography of postmortem infarcted brain tissue showed focal uptake corresponding to intraluminal thrombus within the culprit vessel and downstream microvasculature. There was also evidence of diffuse uptake within the parenchyma of the infarcted brain tissue reflecting petechial hemorrhage (Figure S2). There was no uptake of ^18^F-GP1 seen in either control samples or remote noninfarcted brain tissue from patients who had sustained a stroke (Figure S1).

## DISCUSSION

In this proof-of-concept study, we performed a prospective assessment of ^18^F-GP1 PET and CT angiography to identify the presence of thrombus in patients with acute stroke. We have demonstrated its ability to identify in vivo thrombus in both the vasculature and the brain with major potential to explore the role and origin of thrombosis in the etiology and pathogenesis of stroke.

In this study, we found ^18^F-GP1 uptake in both the carotid arteries and the brain. Carotid artery uptake was focal and mostly unilateral, occurring at sites of atheroma. In all but 1 case, there was no uptake in nonculprit carotid plaques, even in the presence of severe stenosis. This suggests ^18^F-GP1 uptake is not directly associated with the presence of atheroma or degree of stenosis and could indicate the presence of activated platelets after recent atherosclerotic plaque rupture or erosion. Of the 2 participants without ^18^F-GP1 uptake on culprit carotid plaque, 1 showed uptake around an aortic valve replacement (patient number 4 in Table S1) and the other showed unilateral carotid ^18^F-GP1 uptake in the contralateral vessel, which contained a previously implanted internal carotid artery stent (patient number 5 in Table S1). The remaining cases without carotid artery uptake were a posterior circulatory stroke and 2 cases that were thought to be cardioembolic. We would not expect to find carotid artery uptake in these cases.

Brain uptake of ^18^F-GP1 was seen in all areas of acute CT and magnetic resonance imaging–defined infarction. One case showed uptake within the corresponding area to stroke symptoms with an acute small cortical infarct on CT. This suggests ^18^F-GP1 PET– CT imaging can highlight small acute infarcts that may be difficult to visualize on unenhanced CT.

Two patterns of brain uptake were apparent in this limited case series. The first pattern of brain ^18^F-GP1 uptake was visible throughout the affected brain tissue and observed in the patients with cortical infarctions seen within the frontal and parietal lobes, as well as the cerebellum. Qualitatively, the uptake encompassed and colocalized with the wider area of infarction. This appears to correspond to the diffuse uptake of ^18^F-GP1 seen in our ex vivo work, which represented petechial hemorrhage. This likely reflects the loss of integrity of the cerebral microvasculature and blood-brain barrier with activated platelets playing a role in achieving hemostasis and limiting blood extravasation. In a different patient group with acute myocardial infarction, we have found a similar pattern of ^18^F-GP1 uptake within infarcted myocardium indicative of intramyocardial hemorrhage or microvascular obstruction.^15^ Microvascular obstruction following ischemia leading to the no-reflow phenomenon is well described in the context of myocardial infarction and cardiac reperfusion therapy^16^ and is also believed to play a role in cerebral ischemia particularly as thrombolysis and thrombectomy become more widespread.^17^ The disruption of the microvasculature following ischemic stroke is partly caused by narrowed capillary lumens, which contain entrapped fibrin-platelet deposits and other blood components.^18^ The diffuse uptake of ^18^F-GP1 in vivo as well as representing diffuse parenchymal petechial hemorrhage could represent microvascular obstruction. Our ex vivo histology data of infarcted brain tissue support these possibilities. ^18^F-GP1 uptake correlates with the specific CD41 (platelet glycoprotein IIb) immunohistochemistry, which targets the same platelet glycoprotein IIb/IIIa receptor as ^18^F-GP1. Moreover, ^18^F-GP1 uptake is blocked by the unlabeled GP1 reference standard (Figure; Figure S2) demonstrating that there is no nonspecific binding. This has previously been demonstrated in several other tissues including lung parenchyma,^19^ bioprosthetic aortic valves,^4^ and coronary thrombectomy specimens.^5^ However, we should acknowledge that there may be some nonspecific uptake of the tracer within the infarcted region although we were unable to demonstrate nonspecific uptake in our ex vivo postmortem studies of infarcted brain tissue.

The second pattern of brain ^18^F-GP1 uptake was more focal and was seen in areas adjacent to the regions of infarction, with both index cases occurring within the deep structures of the brain in the left basal ganglia and left corona radiata. In our ex vivo work, we have seen focal uptake of ^18^F-GP1 corresponding to occluded thrombosed small vessels, as well as partially occluded vessels with intraluminal thrombus. We previously described thrombus in the perforating arteriole lumen or vessel wall in recent small subcortical (lacunar) ischemic stroke on magnetic resonance and CT imaging, occurring in about 10% of cases.^20^ Therefore, these focal areas seen on imaging may represent intraluminal thrombus within the small perforating arterioles. The intense uptake appears larger than expected due to the partial volume effects that overestimate the true size of the underlying lesion seen with the limited resolution of PET.

In some stroke subtypes, there remains uncertainty as to the presence and contribution of thrombus, such as strokes caused by small vessel disease. One case demonstrated focal brain ^18^F-GP1 uptake around a small subcortical infarction with associated bilateral carotid artery uptake (patient number 10 in Table S1), which could suggest carotid thromboembolism as a potential cause of this lacunar stroke, known to occur in about 15% of lacunar strokes. It is also relevant in cryptogenic stroke, which accounts for 1 in 6 ischemic strokes and where patients have a high stroke reoccurrence rate of ≈25% at 5 years.^21^ Two of the 4 cases with undetermined cause of stroke showed bilateral carotid uptake, which again would suggest atherothromboembolism as the cause of stroke despite the absence of stenotic (>50%) carotid atheroma. Overall within this pilot cohort, ^18^F-GP1 imaging suggested an additional mechanism of stroke in 3 of the 11 cases. Thus, ^18^F-GP1 PET and CT angiography may have the potential to improve our understanding of these stroke subtypes and allow more targeted antithrombotic interventions.

It is important to speculate on the potential clinical utility of ^18^F-GP1 PET and CT angiography in patients with stroke or transient ischemic attack. Two of the 5 participants undergoing carotid endarterectomy did not have ipsilateral culprit carotid artery ^18^F-GP1 uptake, with 1 having a potential cardiac source of thromboembolism. Conversely, 2 participants were found to have carotid ^18^F-GP1 uptake where either the origin of the thromboembolism was uncertain or had been attributed to small vessel disease. Finally, 2 further cases were informed by the presence or absence of thrombosis: one identifying cerebral thrombosis in a patient with a small acute cortical infarct on brain CT that might easily be overlooked and another with cryptogenic recurrent stroke where no evidence of thrombosis was found. In the latter case, the patient had undergone percutaneous patent foramen ovale closure because of recurrent presumed thromboembolic stroke. The absence of any ^18^F-GP1 uptake within the vasculature suggests a nonthromboembolic cause of these cryptogenic strokes, the etiology of which has yet to be defined, perhaps indicating in situ thrombosis. Thus, we have found that ^18^F-GP1 PET and CT angiography provided important and potentially clinically relevant findings in a high proportion of this preliminary case series and have the potential to assist in the identification of the role and origin of thrombosis in patients with ischemic stroke. Indeed, we have recently demonstrated the potential for ^18^F-GP1 PET and CT angiography was able to identify left atrial appendage thrombus, which led to the reclassification and treatment of a patient with acute myocardial infarction.^6^

We acknowledge several limitations of this preliminary case series. Our descriptive findings are limited by their preliminary nature, small sample size, and broad heterogeneity of enrolled patients. In addition, the PET and CT did not routinely include the entire heart, which meant it was not possible to assess systematically for cardiogenic sources of stroke. We have also yet to establish the repeatability and reproducibility of ^18^F-GP1 imaging in the vasculature and the brain. Our proof-of-concept data should only act as a platform to inform further evaluation and prospective clinical studies of this exciting imaging approach. We acknowledge much of our interpretation by necessity is speculative and requires a larger cohort study (TORPIS study [The Origin and Role of Thromboembolism in the Pathogenesis of Ischaemic Stroke]; https://www.clinicaltrials.gov; NCT05636748).

In conclusion, ^18^F-GP1 PET and CT angiography can noninvasively detect in vivo thrombus in the vasculature and brain of patients with acute ischemic stroke and transient ischemic attacks. This has the potential to help define the role and origin of thrombus in patients with stroke and may in the future direct the most appropriate therapy and interventions to prevent recurrent stroke.

## ARTICLE INFORMATION

### Sources of Funding

B. Whittington (FS/CRTF/21/24129), E. Tzolos (FS/CRTF/20/24086), N.L. Mills (CH/F/21/90010 and RG/20/10/34966), M.C. Williams (FS/ICRF/20/26002, FS/11/014, and CH/09/002), D.E. Newby (CH/09/002, RG/16/10/32375, and RE/18/5/34216), and M.R. Dweck (FS/14/78/31020) are supported by the British Heart Foundation. D.E. Newby is the recipient of a Welcome Trust Senior Investigator Award (WT103782AIA). E.J.R. van Beek is supported by the Scottish Imaging Network: a Platform of Scientific Excellence. M.R. Dweck is supported by the Sir Jules Thorn Biomedical Research Award 2015 (15/JTA). FusionQuant analysis tools and P.J. Slomka are supported by the National Institutes of Health (NIH) National Heart, Lung, and Blood Institute (NHLBI) grant 5R01HL135557. D. Dey is supported by the NIH NHLBI grant 1R01HL148787-01A1. W. Whiteley is supported by the Chief Scientist’s Office, Alzheimer’s Society. J.M. Wardlaw is supported by the UK Dementia Research Institute, which receives its funding from DRI, Ltd, funded by the UK Medical Research Council, Alzheimer’s Society and Alzheimer’s Research UK. The Edinburgh Clinical Research Facilities, Edinburgh Imaging Facility, and Edinburgh Clinical Trials Unit are supported by the National Health Service Research Scotland through the National Health Service Lothian Health Board. For the purpose of open access, the author has applied a Creative Commons Attribution (CC-BY) licence to any author accepted manuscript version arising from this submission.

### Disclosures

N. Koglin and A.W. Stephens are employees of Life Molecular Imaging, who provided reagents for ^18^F-GP1 production. The other authors report no conflicts.

### Supplemental Material

Supplemental Methods

Supplemental Results

Table S1

Figures S1–S2

Major Resource Table

## Supplementary Material


