# Supplementary figures and images for: Non-invasive In Vivo Thrombus Imaging in Patients with Ischemic Stroke or Transient Ischemic Attack
Source: Arterioscler Thromb Vasc Biol. Author manuscript; Available in PMC 2023 Aug 23. (PMC10443628; doi:10.1161/ATVBAHA.122.318204)

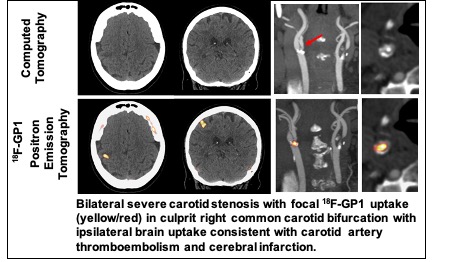

Supplement: Graphic Abstract [file EMS178542-supplement-Graphic_Abstract.jpg]
