# Supplementary material for: Non-invasive In Vivo Thrombus Imaging in Patients with Ischemic Stroke or Transient Ischemic Attack
Source: Arterioscler Thromb Vasc Biol. Author manuscript; Available in PMC 2023 Aug 23. (PMC10443628; doi:10.1161/ATVBAHA.122.318204)
Supplement: Supplementary Materials [file EMS178542-supplement-Supplementary_Materials.pdf]

Supplementary Material

Non-invasive In Vivo Thrombus Imaging in Patients with Ischemic Stroke or Transient Ischemic Attack

Beth Whittington MD,[a] Evangelos Tzolos MD,[a] Rong Bing MBBS PhD,[a] Jack Andrews MD,[a] Christophe Lucatelli PhD,[b] Mark G MacAskill PhD,[a, b] Adriana A. S. Tavares PhD,[a, b] Tim Clark BSc MSc Dip IPEM(S),[b] Nicholas L. Mills MD PhD,[a, d] Jennifer Nash MD,[a] Damini Dey PhD,[f] Piotr J. Slomka PhD,[f] Norman Koglin PhD,[e] Andrew W. Stephens MD PhD,[e] Edwin J. R. van Beek MD PhD,[a, b] Colin Smith MD PhD[g], Marc R. Dweck MD PhD,[a, b] Michelle C. Williams MBChB PhD,[a, b] William Whiteley MD PhD,[c] Joanna M Wardlaw MD PhD,[c,h] David E. Newby MD PhD[a, b]

[a]BHF Centre for Cardiovascular Science, University of Edinburgh, Edinburgh,
[b]Edinburgh Imaging, Queen's Medical Research Institute, Edinburgh, UK
[c]Centre for Clinical Brain Sciences, University of Edinburgh, UK
[d]Usher Institute, University of Edinburgh, Edinburgh, UK
[e]Life Molecular Imaging GmbH, Berlin, Germany
[f]Departments of Medicine (Division of Artificial Intelligence in Medicine) and Biomedical Imaging Research Institute, Cedars-Sinai Medical Centre, Los Angeles, USA
[g] Division of Pathology, University of Edinburgh, Teviot Place, UK.
[h]UK Dementia Research Institute Centre at the University of Edinburgh

Correspondence address
Dr Beth Whittington
BHF Centre for Cardiovascular Science, University of Edinburgh
47 Little France Crescent
Edinburgh EH16 4TJ
Email: b.whittin@ed.ac.uk

Supplementary Materials

I. Supplementary Methods

II. Supplementary Results

III. Supplementary Tables

IV. Supplementary Figures S1-S2

V. Major Resource Table

Supplementary Methods

^{18}F-GP1 Radiosynthesis

Radiosynthesis of ^{18}F-GP1 was carried out using a NEPTIS Perform synthesizer (Optimised Radiochemical Applications, Neuville, Belgium). The synthesis is a two-step process, involving the nucleophilic fluorination of the tosylate precursor (GP1 precursor) followed by the removal of the protecting groups. After neutralization and separation by semi-preparative high-performance liquid chromatography, ^{18}F-GP1 was then formulated in phosphate buffer before being terminally sterilized in an autoclave.

Ex-vivo thrombus studies in brain tissue

Brain samples were obtained from the Edinburgh Brain Bank. These included post-mortem sections of infarcted brain tissue from four cases of patients that had died as a result of ischemic stroke. In these cases, contralateral brain samples were also obtained of non-infarcted areas contralateral to the area of infarction. Five samples of brain tissue from control patients without ischemic stroke were also obtained. These samples were obtained from patients who had not undergone the *in vivo* positron emission tomography imaging.

^{18}F-GP1 Autoradiography

Autoradiography was performed on brain tissue samples (n=12). Two paraffin embedded sections (5 µm) were de-paraffinized and sections were incubated with 20 nM ^{18}F-GP1 in phosphate buffered saline for 60 min at room temperature, with and without 10 µM unlabeled GP1 as a blocking agent. After two 5 min washes in phosphate buffered saline and one in deionized water, the slides were dried and exposed to the autoradiography screen (BAS-IP-SR 2040, Cytiva, USA) overnight. The screens were visualised on an Amersham Typhoon IP

Biomolecular Imager (Cytiva, USA) with the standardised “S4000” voltage setting at a 10 µm resolution.

Histology

Immunohistochemistry was completed for CD41 with a rabbit monoclonal [EPR4330] antibody to CD41 (Abcam, Cat no: ab134131) at a dilution of 1:750. Staining was performed via automated staining with a Leica Bond Rx system using Bond Epitope Retrieval Solution 2 (pH=9, Catalog No: AR9640) and Bond Polymer Refine Detection DAB (Catalog No: DS9800). The omission of the primary antibody served as negative controls. High-resolution images were generated using an Aperio Slide Scanner and ImageScope software (Leica Biosystems, Germany).

Hematoxylin and eosin staining was performed on 4-µm paraffin sections of the brain tissue specimens.

Supplementary Results

In patients suffering a transient ischemic attack or ischemic stroke thought to be due to large artery atheroembolism from carotid artery disease, 60% had vascular ^{18}F-GP1 uptake on the culprit carotid artery. Of those patients that did not show vascular uptake on the culprit carotid artery they had a median time of symptom onset to imaging of 11 days compared to 6 days in those that uptake on the culprit carotid plaque. In patients with an ischemic infarction on computed tomography or magnetic resonance imaging, all cases had brain ^{18}F-GP1 uptake.

Supplementary Tables

Table S1. Clinical characteristics and image findings.

#	Age/ Gender	Presenting Neurological Symptoms	Clinical Diagnosis as per OSCP	CT brain at presenting	GP1 uptake in brain	GP1 uptake in Carotid Arteries	Time from Symptoms Onset to PET-CT imaging (days)	Under-went CEA	Potential cause as per TOAST criteria	Does the 18F-GP1 suggest alternative mechanism of stroke?
1	79 Female	Left arm weakness	Right LACI	Moderate small vessel change, no acute intracranial pathology	Right posterior frontal cortex	Yes- Right (culprit)	2	Yes	Large artery atherosclerosis	No
2	71 Female	Left arm weakness	Right Hemisphere LACI	Focal subacute frontal lobe infarction	Right frontal lobe	Yes- Right (culprit)	14	Yes	Large artery atherosclerosis – carotid atheroembolism	No
3	75 Male	Recurrent loss of vision right eye (amaurosis fugax)	Right retinal TIA	No acute infarction.	No uptake	Yes- Right (culprit)	11	Yes	Large artery atherosclerosis – carotid atheroembolism	No
4	63 Male	Slurred speech, left arm weakness, confusion	Right Hemisphere PACI	Old left frontal and parietal cortical infarctions. No acute intracranial pathology	No uptake	Yes-Left non culprit (ICA stent on contralateral side)	6	Yes	Large artery atherosclerosis – carotid atheroembolism	No

#	Age/ Gender	Presenting Neurological Symptoms	Clinical Diagnosis as per OSCP	CT brain at presenting	GP1 uptake in brain	GP1 uptake in Carotid Arteries	Time from Symptoms Onset to PET-CT imaging (days)	Under-went CEA	Potential cause as per TOAST criteria	Does the 18F-GP1 suggest alternative mechanism of stroke?
5	86 Male	Visual loss right eye	Right central retinal artery occlusion	No acute intracranial pathology	No uptake	No uptake	11	Yes	Large artery atherosclerosis – carotid atheroembolism	Yes- Uptake around previous aortic valve replacement indicating possible cardioembolic, no uptake seen on culprit carotid artery
6	49 Male	Double vision, Left facial droop, dysarthria, ataxia	Right POCI	Subacute right cerebellar infarction	Right cerebellum	No	11	No	Undetermined – incomplete investigations	No
7	84 Male	Visual loss, speech disturbance, right arm/leg weakness	Left TACI	No evidence of acute intracranial pathology	No uptake	Yes - bilateral	2	No	Undetermined cause- incomplete investigations	Yes- carotid uptake suggesting possible atheroembolism
8	103 Male	Right arm and leg weakness, reduced GCS	Left PACI	Diffuse global atrophy old right sided caudate lacunar infarction. No acute intracranial pathology on standard or	Left Basal ganglia	No	4	No	Possible cardioembolic (subtherapeutic INR with permanent atrial fibrillation)	No

#	Age/ Gender	Presenting Neurological Symptoms	Clinical Diagnosis as per OSCP	CT brain at presenting	GP1 uptake in brain	GP1 uptake in Carotid Arteries	Time from Symptoms Onset to PET-CT imaging (days)	Under-went CEA	Potential cause as per TOAST criteria	Does the 18F-GP1 suggest alternative mechanism of stroke?
				care CT. Research CT shows evolving left basal ganglia infarction.						
9	79 Female	Right arm and leg weakness	Left LACS	Patchy small vessel change. No acute intracranial findings MRI- acute left parietal infarction	Left parietal cortical	No	4	No	Potentially cardioembolic – repeated cortical infarctions suggest embolic source proximal to neck arteries	No
10	74 Male	Right upper limb weakness	Left LACI	Left Subacute corona radiata infarct	Left corona radiata	Yes-bilateral	21	No	Undetermined due competing causes-likely small vessel disease on CT scan but some large vessel atheroma present bilateral carotid bifurcation but worse on left	Yes- carotid uptake suggesting possible atheroembolism as opposed to small vessel disease
11	70 Male	Left sided facial droop, dysarthria	Right sided TIA	Old bilateral infarctions – left occipital	No uptake	Yes- Left	19	No	Undetermined due to	No

#	Age/ Gender	Presenting Neurological Symptoms	Clinical Diagnosis as per OSCP	CT brain at presenting	GP1 uptake in brain	GP1 uptake in Carotid Arteries	Time from Symptoms Onset to PET-CT imaging (days)	Under-went CEA	Potential cause as per TOAST criteria	Does the 18F-GP1 suggest alternative mechanism of stroke?
				lobe and right temporoparietal junction. No acute findings.					incomplete investigations	

The Oxfordshire Community Stroke Project (OSCP), Lacunar infarction (LACI), Partial anterior circulation infarction (PACI), Total anterior circulation infarction (TACI), Posterior circulation infarction (POCI), Transient Ischemic Attack (TI), Positron emission tomography (PET) Computed tomography (CT), Carotid Endarterectomy (CEA), International normalized ratio. (INR).

Supplementary Figures

Figure S1. ^{18}F-GP1 uptake in ex-vivo non infarcted brain tissue.

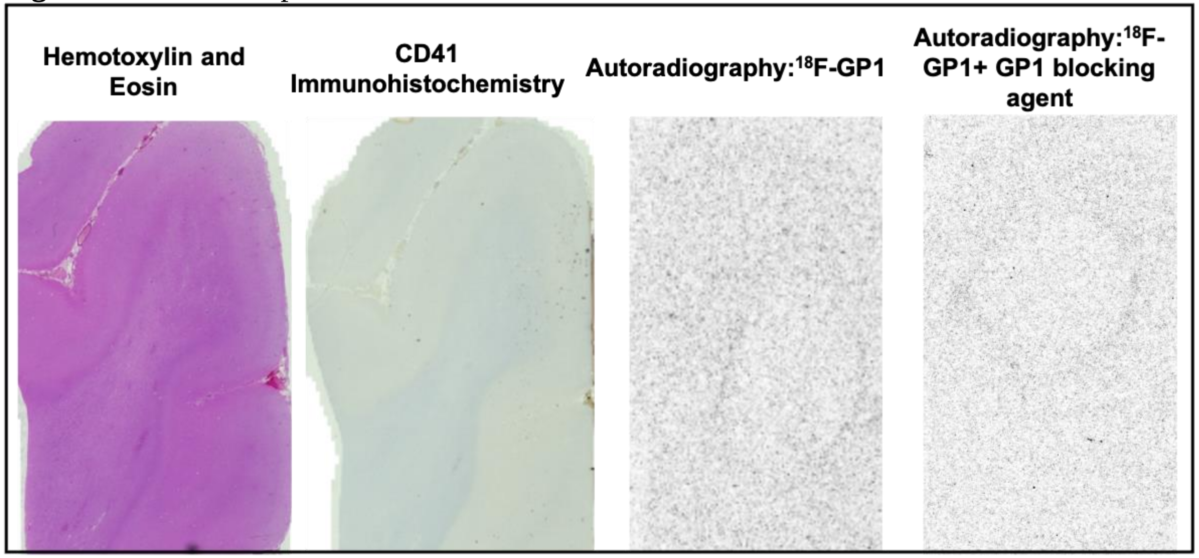

Figure S1.Post mortem remote non-infarcted brain tissue (contralateral to area of infarction) in patients following acute middle cerebral artery territory ischemic stroke. Hemotoxylin and Eosin (H&E) staining showing area of non-infarcted tissue with no significant CD41 staining and no ^{18}F-GP1 uptake on autoradiography.

Figure S2. Diffuse ^{18}F-GP1 uptake in ex-vivo post mortem infarcted brain tissue.

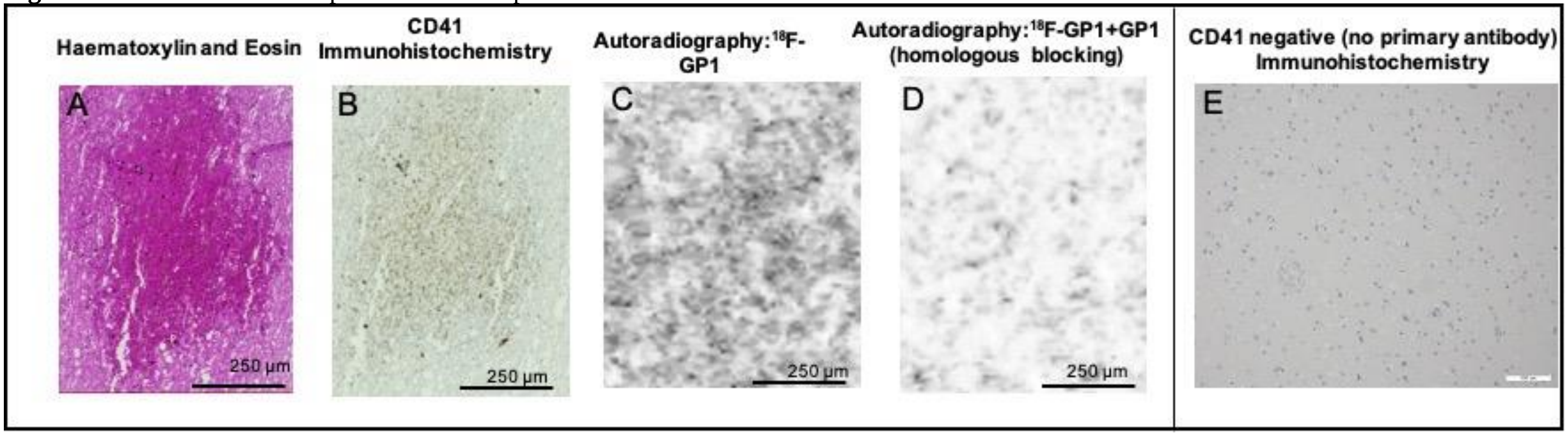

Figure S2. Post-mortem infarcted brain tissue. Area of petechial hemorrhage accentuated on Haematoxylin and Eosin (H&E) staining (A) with CD41 platelet immunostaining (B). Corresponding diffuse parenchymal uptake of ^{18}F-GP1 (C) which is blocked by the co-administration of GP1 reference standard indicating specific binding (D). (E) Negative CD41 staining (no primary antibody) of different non infarcted brain tissue sample.

Major Resources Table

Animals (in vivo studies)

Species	Vendor or Source	Background Strain	Sex	Persistent ID / URL
NA				

Genetically Modified Animals

	Species	Vendor or Source	Background Strain	Other Information	Persistent ID / URL
**Parent - Male**	NA				
**Parent - Female**	NA				

Antibodies

Target antigen	Vendor or Source	Catalog #	Working concentration	Lot # (preferred but not required)	Persistent ID / URL
NA					

DNA/cDNA Clones

Clone Name	Sequence	Source / Repository	Persistent ID / URL
NA			

Cultured Cells

Name	Vendor or Source	Sex (F, M, or unknown)	Persistent ID / URL
NA			

Data & Code Availability

Description	Source / Repository	Persistent ID / URL
NA		

Other

Description	Source / Repository	Persistent ID / URL
NA		
